# Regulation of *Cunninghamella* spp. biofilm growth by tryptophol and tyrosol

**DOI:** 10.1016/j.bioflm.2021.100046

**Published:** 2021-03-26

**Authors:** Mohd Faheem Khan, Dua Saleem, Cormac D. Murphy

**Affiliations:** UCD School of Biomolecular and Biomedical Science, University College Dublin, Belfield, Dublin 4, Ireland

**Keywords:** *Cunninghamella*, Filamentous fungus biofilm, Quorum sensing, 3-hydroxytyrosol, Tryptophol, Tyrosol

## Abstract

Fungi belonging to the genus *Cunninghamella* are often used as microbial models of mammalian metabolism owing to their ability to transform a range of xenobiotic compounds. Furthermore, under specific growth conditions species such as *Cunninghamellaelegans* and *Cunninghamellaechinulata* grow as biofilms enabling a convenient semi-continuous production of valuable drug metabolites. However, the molecular mechanism of biofilm regulation is not understood, thus controlling biofilm thickness limits the productive applications of it. In this paper we describe the identification of two molecules, tyrosol and tryptophol, that were identified in *C. blakesleeana* cultures, but not in *C. elegans* and *C. echinulata*. The molecules are known quorum sensing molecules (QSMs) in yeast and their potential role in *Cunninghamella* biofilm regulation was explored. Both were present in higher concentrations in *C. blakesleeana* planktonic cultures compared with biofilms; they inhibited the growth of the fungus on agar plates and selectively inhibited biofilm growth in liquid cultures. The molecules had a comparatively minor impact on the biofilm growth of *C. elegans* and *C. echinulata* and on the growth of these fungi on agar plates. Finally, when exogenous tyrosol or tryptophol was added to previously grown *C. blakesleeana* biofilm, detachment was visible and new additional planktonic culture was measured, confirming that these molecules specifically regulate biofilm growth in this fungus.

## Highlights

• Tyrosol and tryptophol were identified in culture supernatants of *Cunninghamella* blakesleeana.
• Concentrations of the compounds were substantially higher in planktonic cultures compared with biofilms.
• Bioassays revealed that tyrosol and tryptophol inhibited growth of *C. blakesleeana* on agar plates.
• Biofilm growth was inhibited by exogenous addition of the compounds whereas planktonic growth was unaffected.
• The compounds caused detachment of previously grown biofilms.

## Graphical abstract

**Figure:**
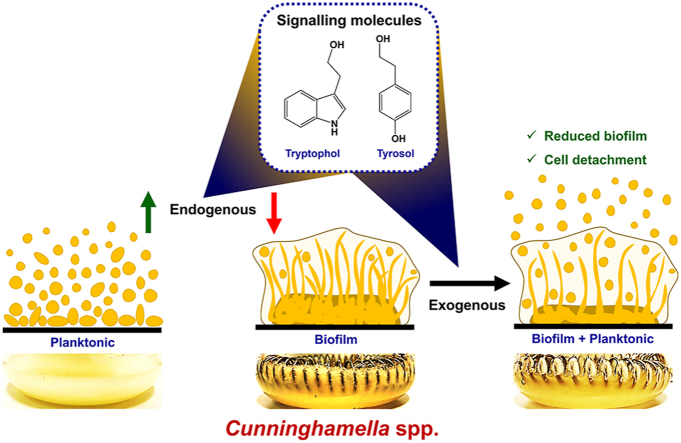

## Introduction

Although much of the focus in biofilm research has been on controlling growth to prevent fouling and infection, there is increasing recognition that biofilms are biotechnologically relevant [12,18]. Compared to the planktonic culture, biofilms are more stable, can tolerate the presence of toxic compounds, are easily reused and are readily separated from the culture supernatant where the valuable enzymes and metabolites usually are, simplifying product purification. Although filamentous fungi are employed for a range of biotechnological purposes, such as enzyme and antibiotic production, research on biofilms in these applications is relatively limited. Nevertheless, there is interest in the ability of filamentous fungal biofilms to enhance enzyme production [3], dye decolourisation [20] and metabolite biosynthesis [30].

Fungi belonging to the genus *Cunninghamella*, in particular *C. elegans*, *C. blakesleeana* and *C. echinulata*, are often used as models of mammalian xenobiotic metabolism owing to their ability to generate phase I (oxidative) and phase II (conjugative) metabolites when incubated with a range of non-natural compounds [21,23]. The fungi have prominent cytochrome P450 activity, which accounts for their ability to oxidatively catabolise xenobiotics [22]. Furthermore, *Cunninghamella* spp. can grow as biofilms when cultivated under specific conditions [1,7,19,29]. These biofilms are more productive than planktonic cultures for the generation of valuable drug metabolites since they can be repeatedly used for biotransformations and the supernatant is easily separated from the biomass [24]. Furthermore, the biofilm culture is more effective at removing dye and metal ions from water compared with the planktonically-grown fungus [16].

One issue with using biofilms for drug metabolite production is that excessive biomass can potentially limit the recovery of the metabolite, since increased biofilm thickness results in a lower volume of supernatant that is available for extraction [1]. In order to better control biofilm growth, the molecular mechanism used by the fungus to regulate this mode of growth needs to be understood. Until very recently, nothing was known about how the fungus regulates the growth of biofilm; Khan and Murphy [17] identified 3-hydroxytyrosol as a novel regulator molecule biosynthesised by *C. elegans*, which is produced in greater amounts in planktonic cultures and selectively inhibits biofilm growth. Prompted by this discovery we examined the production of similar molecules in the related fungi *C. echinulata* and *C. blakesleeana* and investigated their effects on biofilm and planktonic growth.

## Materials and methods

### Chemicals, fungi and culturing conditions

Tyrosol, 3-hydroxytyrosol, tryptophol (3-indoleethanol), N-methyl-N-(trimethylsilyl)trifluoroacetamide (MSTFA), Sabouraud dextrose broth (SDB) and Sabouraud 4% dextrose agar (SDA) were procured from Sigma-Aldrich (Arklow, Ireland). Solvents and other reagents used in the study were of analytical grade.

Three species of *Cunnighamella* (*C. echinulata* DSM1905, *C. blakesleeana* DSM1906 and *C. elegans* DSM1908) were used in the present study and purchased from DSMZ - German Collection of Microorganisms and Cell Cultures GmbH. The procedure for fungal cultivation is the same as described in Ref. [1]. Briefly, the fungal inocula were prepared by homogenisation of grown mycelium of *Cunnighamella* sp. (streaked on SDA plates and incubated at 28 ᵒC for 5 days) with 100 ​mL of autoclaved deionised water. Both biofilm and planktonic cultures were grown in 50 ​mL SDB in 250-mL conical flasks with 1 ​mL and 5 ​mL inoculum, respectively. The biofilms were only grown in flask fitted with spring (T316 stainless steel wire, 1.2 ​mm thickness).

### Identification and quantification of putative signalling molecules using gas chromatography-mass spectrometry

*C. blakesleeana* DSM1906 planktonic and biofilm cultures were incubated for up to 240 ​h. The supernatants of planktonic cultures were separated by centrifugation at 8000 ​rpm for 10 ​min (Thermo Scientific SL 8R centrifuge fitted with a HIGHConic III fixed angle rotor), whereas the supernatants of biofilm cultures were easily decanted from the flask as the fungi remained adhered to the metal spring. The metabolites produced by both the culture types were extracted from their aqueous fractions using ethyl acetate. The solvent was evaporated under reduced pressure and the residue was re-dissolved in 1 ​mL ethyl acetate. Samples were dried under a stream of N_2_ gas and silylated using MSTFA (80 ​μL) and heating at 100 ​°C for 60 ​min; the final volume was adjusted to 500 ​μL with ethyl acetate. Analysis was conducted on a 7890B N Agilent GC system equipped with HP-5MS column (30 ​m ​× ​0.25 ​mm ​× ​0.33 ​μm) for chromatographic separation coupled to a 5977A mass-selective detector (Agilent Technologies). The oven was set at an initial temperature of 150 ​°C for 3 ​min then the temperature raised to 270 ​°C at 10 ​°C/min. For quantification of tyrosol and tryptophol, a five-point calibration was conducted with authentic standards (0.25–3 ​mg/mL) and 3-hydroxytyrosol (1 ​mg/mL) that was employed as an internal standard (Fig. S1). The peak areas of the extracted metabolites were compared to that of the internal standard to estimate concentration.

### Biotransformation of tryptophol, tyrosol and 3-hydroxytyrosol

The compounds (5 ​mg dissolved in dimethylformamide) were introduced to the 96 h-grown biofilm and planktonic cultures of *C. echinulata*, *C. blakesleeana* and *C. elegans*, which were further incubated at 28 ᵒC with 150 ​rpm agitation for 96 ​h. Their aqueous fractions were collected, metabolites extracted, derivatised and analysed by GC-MS as described above.

### Effect of tyrosol, 3-hydroxytyrosol and tryptophol on *Cunninghamella* spp.

The compounds were dissolved in dimethylformamide (DMF), added to wells in SDA plates (formed with a sterilized borer (diameter ​= ​1 ​cm)) and allowed to stand for 1 ​h to allow diffusion. The plates were inoculated with liquid fungal inoculum (50 ​μL) and incubated at 28 ᵒC for 96 ​h. The effect of the compounds on the fungal growth in liquid cultures was enabled by adding them directly to flasks prior to inoculation. The flasks were incubated for 96 ​h at 28 ᵒC with 150 ​rpm agitation. The biomass was harvested, dried for 24 ​h at 80 ᵒC and weighed. For the control experiments, DMF only was added to cultures.

### Cell detachment experiment

*C. blakesleeana* biofilm cultures were grown for 144 ​h and the culture fluid decanted. The flask was washed with 50 ​mL sterile water and fresh SDB medium containing different amounts of tryptophol and tyrosol was added. The cultures were incubated for 96 ​h. The biofilm biomass on the spring and the biomass in the bulk liquid (planktonic) were separated, dried for 24 ​h at 80 ᵒC and weighed.

### Statistical analysis

All the analyses were carried out in triplicates and stated as mean ​± ​standard deviation (error bars) using OriginPro 8.5. Paired sample *t*-test was employed to determine significant differences between means. The null hypothesis was set as the mean of the fungal dried biomass in the planktonic and biofilm cultures remains unchanged after treatment with tyrosol or tryptophol ; p-value ​< ​0.05 was considered a significant difference .

## Results and discussion

### Tryptophol and tyrosol are produced by *C. blakesleeana*

**Fig. 1:**
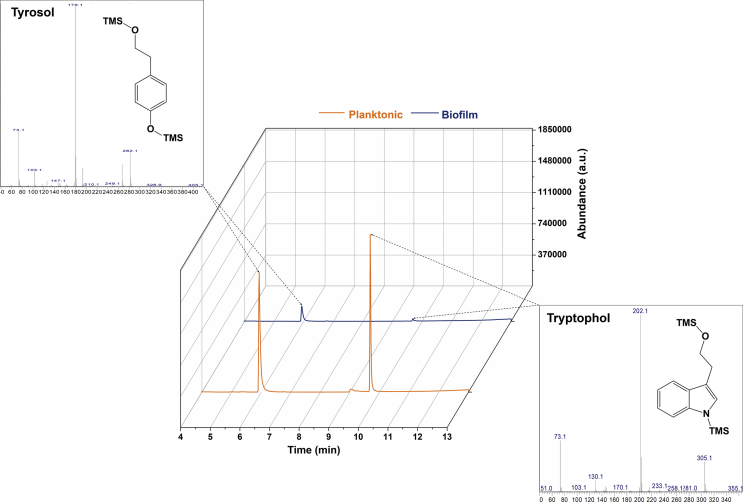
Total ion chromatograms of ethyl acetate extracts from planktonic and biofilm cultures of *C. blakesleeana* grown for 120 ​h showing the presence of tyrosol (retention time 5.9 ​min) and tryptophol (retention time 9.7 ​min). The identities were established from the mass spectra of the analytes and comparison with authentic standards.

**Fig. 2:**
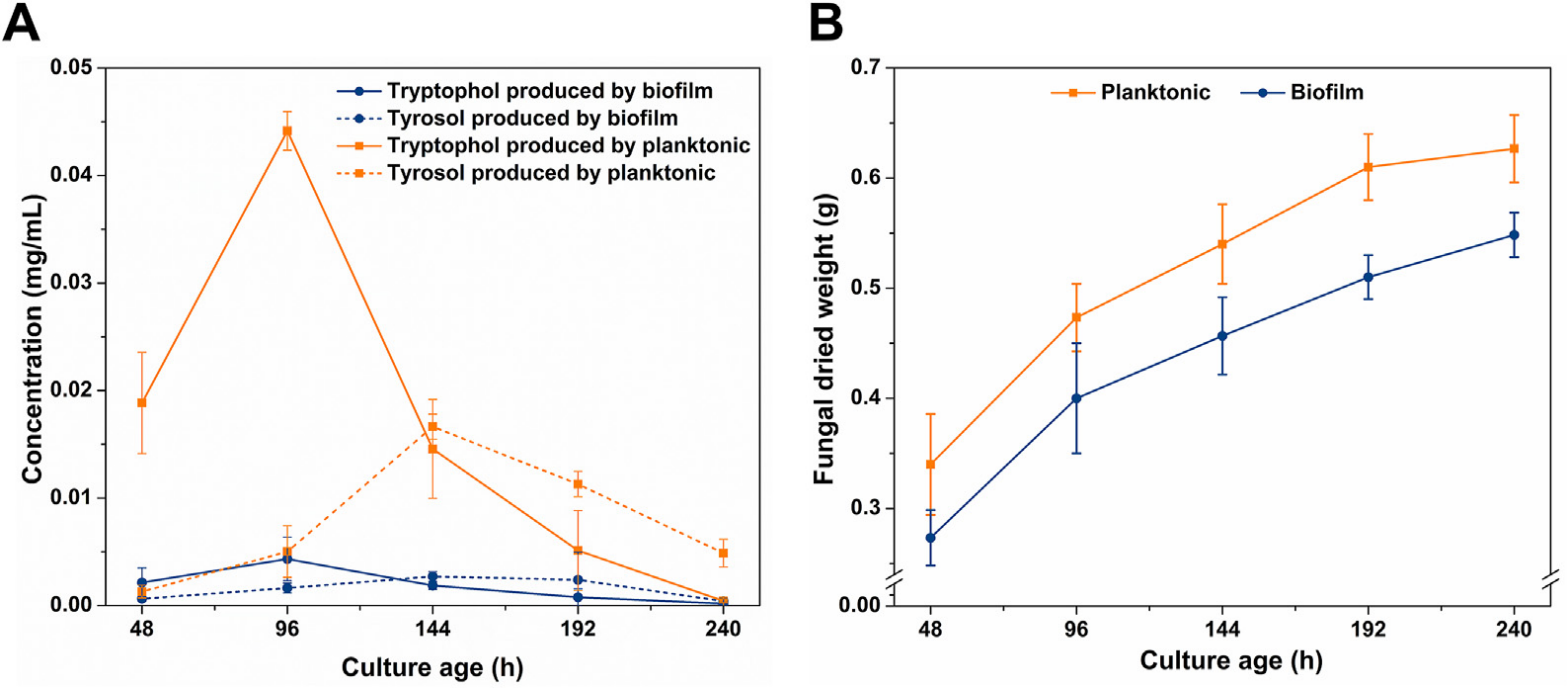
(A) Quantification of tryptophol and tyrosol in planktonic and biofilm cultures of *C. blaskesleeana* over time. Replicate cultures at each time point were extracted with ethyl acetate and samples were analysed by GC-MS. (B) Growth curve of planktonic and biofilm cultures of *C. blaskesleeana*. The dry weight of the biomass was measured in triplicate flasks.

*C. blakesleeana* and *C. echinulata* are two species that are often used in xenobiotic biotransformation studies [4,10,15], and both grow as biofilms if cultivated in the same manner as *C. elegans*, i.e., in an Erlenmeyer flask containing a steel spring. To determine what, if any, potential signalling molecules are produced by these fungi, supernatants from 120 ​h planktonic and biofilm cultures were extracted with ethyl acetate, and the extracts analysed by GC-MS. In *C. blakesleeana*, tryptophol and tyrosol were identified (Fig. 1) by comparing their retention times and mass spectra to authentic standards. In contrast, neither compound was detected in *C. echinulata*, nor was there any 3-hydroxytyrosol produced by this fungus under the conditions used. This molecule was recently identified as a biofilm regulator in *C. elegans* [17]. Both tryptophol and tyrosol are known quorum signalling molecules in other fungi [6,31] but have not been detected in *Cunninghamella* spp. previously. Furthermore, the concentrations detected in the *C. blakelsleeana* cultures appeared dramatically different according to peak heights, with more seemingly present in planktonic cultures (Fig. 1). To confirm this, the production of both compounds was monitored and quantified throughout the growth of the fungus (Fig. 2 and Fig. S2). Tryptophol production peaked at 96 ​h and its concentration was approx. 10.2-fold more in planktonic cultures compared to biofilm. The concentrations decreased dramatically after 144 ​h, but the proportions remained the same in the different cultures. The production of tyrosol was lower than that of tryptophol, and its production peaked after 144 ​h growth. As with tryptophol there was much less tyrosol in biofilm cultures (6.1-fold) compared with planktonic cultures. Both compounds were produced as the fungus was actively growing (Fig. 2B), thus are not secondary metabolites, the production of which characteristically starts in the late exponential phase and continues into the stationary phase.

The reduction in concentration of both compounds in late *C. blakesleeana* cultures might be due to the cytochrome P450 (CYP) activity that is known in this fungus and which is responsible for the biotransformation of a wide range of aromatic compounds [5]. To investigate this, planktonic and biofilm cultures were grown under standard conditions for 96 ​h and exogenous tyrosol, tryptophol and 3-hydroxytyrosol were added to the flasks. These are the conditions that are typically used for CYP-dependent biotransformation of drugs in this fungus [2]. The incubation was continued for a further 96 ​h before the culture supernatants were extracted with ethyl acetate and the extracts analysed by GC-MS to determine the biotransformation products. Minor oxidation products were identified in cultures supplemented with tyrosol and tryptophol and no biotransformation of 3-hydroxytyrosol occurred, but the main compound detected in all experiments was the untransformed substrate (Fig. S3). Biotransformation experiments were also conducted with *C. elegans* and *C. echinulata* also showing very limited biotransformation of the compounds. Thus, the CYP activity of the fungi is probably not responsible for the degradation of the signalling molecules in growing cultures; the compounds are most likely taken up by the cells and degraded along other endogenous catabolic pathways.

### Tryptophol and tyrosol selectively inhibit biofilm growth

**Fig. 3:**
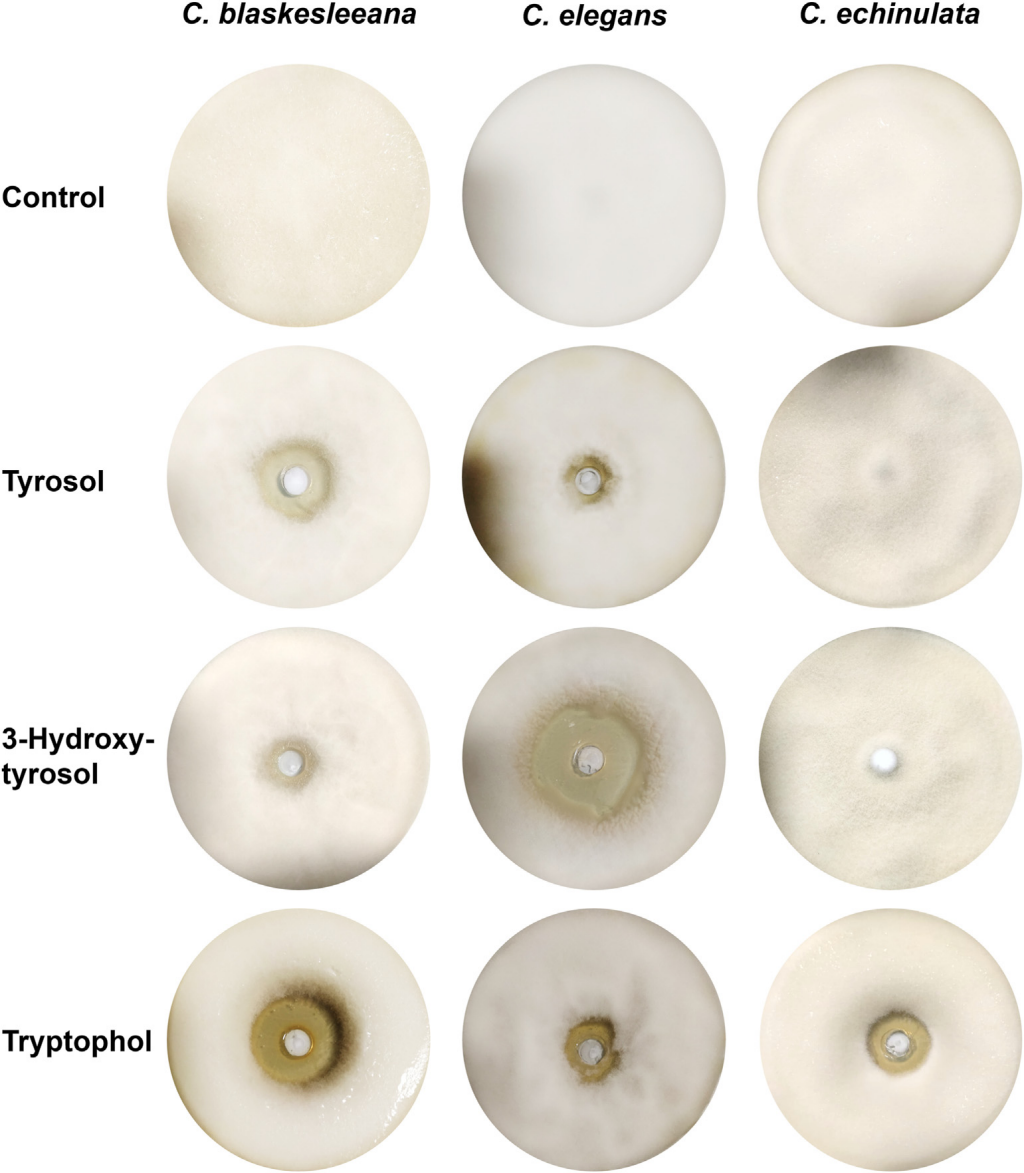
The effect of tyrosol, 3-hydroxytyrosol and tryptophol (5 ​mg) on fungi grown on agar plates. The compounds were dissolved in DMF before adding them to wells in the agar. In the control experiment DMF only was added to the well. Plates were incubated for 96 ​h.

The pattern of production, the difference in the concentrations in biofilm and planktonic cultures and the fact that these compounds have been previously identified as signalling molecules in other fungi suggested a potential role as biofilm regulators, similar to 3-hydroxytyrosol in *C. elegans*. To investigate this, bioassays were established to investigate the inhibitory properties of tyrosol and tryptophol against *C. blakesleeana* when grown on an agar plate. Both molecules resulted in growth inhibition (Fig. 3, Fig. S4-S6), but it was more pronounced with tryptophol. For comparison, *C. echinulata* and *C. elegans* were exposed to the compounds, and inhibition of both fungi by tryptophol was observed, but only *C. elegans* was sensitive to tyrosol. Interestingly, 3-hydroxytyrosol, which is produced by *C. elegans* and strongly inhibits this fungus on agar plates [17], has only a minor effect on the growth of *C. blaskesleeana* on agar plates and no effect on *C. echinulata*.

**Fig. 4:**
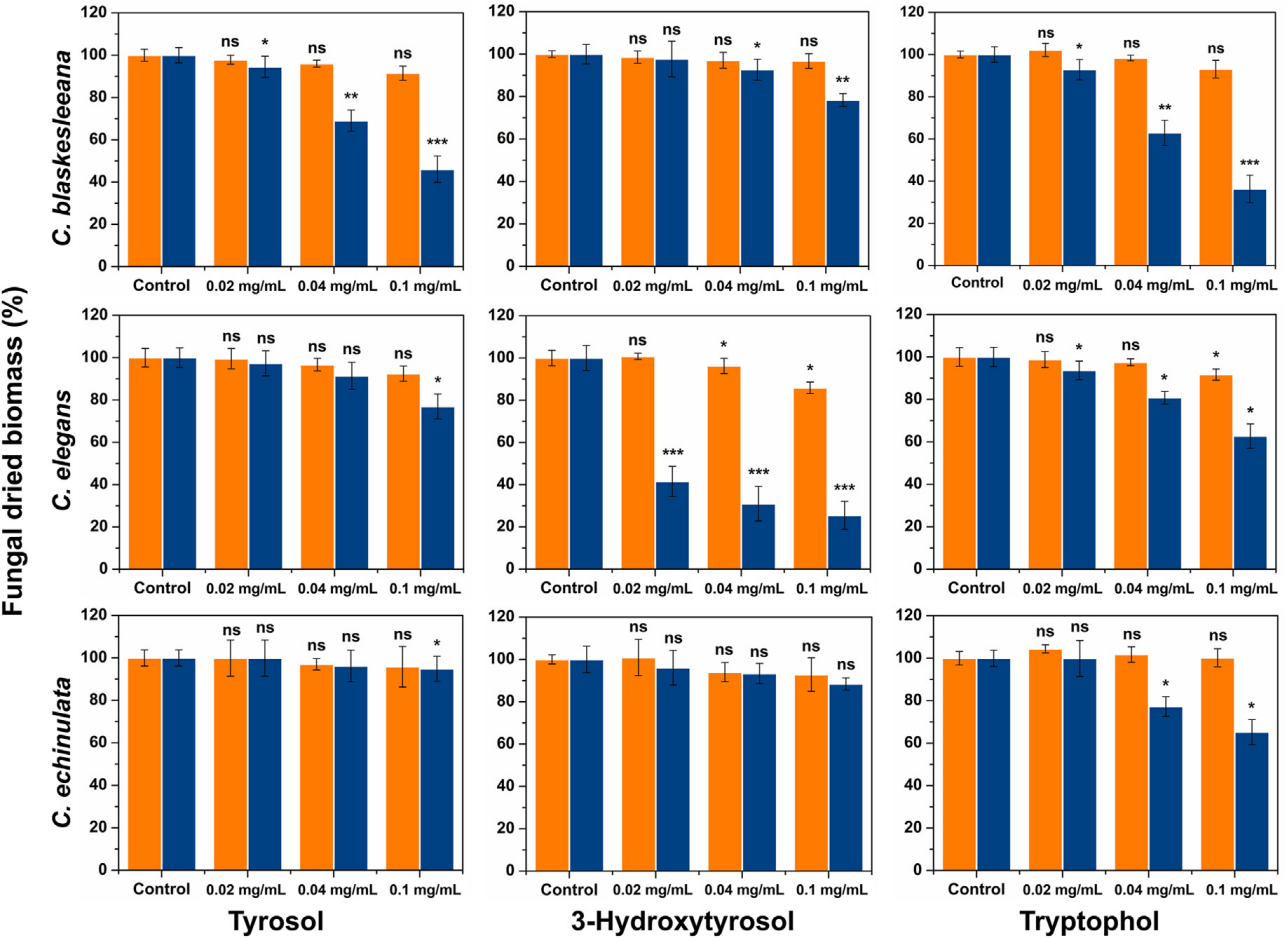
The effect of tyrosol, 3-hydroxytyrosol and tryptophol on the growth of planktonic (orange bars) and biofilm (blue bars) cultures. The growth was measured as dried biomass from triplicate flasks and expressed as an average percentage of that in control cultures to which no compound was added (p-values: >0.05, ns; <0.05, ∗; <0.01, ∗∗; and <0.005, ∗∗∗). The compounds were dissolved in DMF prior to addition to the cultures; DMF only was added to the control flasks. Tryptophol and tyrosol cause detachment of *C. blakesleeana* biofilm. (For interpretation of the references to colour in this figure legend, the reader is referred to the Web version of this article.)

To determine the impact of tyrosol, tryptophol and 3-hydroxytyrosol on the planktonic and biofilm growth of the fungi, the compounds were added to cultures at the start of incubation and the growth measured as dried biomass. Fig. 4 shows that the biofilm growth of all three species was inhibited by the presence of tryptophol, and that the degree of inhibition was greatest in *C. blakesleeana*. Tyrosol also inhibited *C. blakesleeana* biofilm growth at all concentrations tested, whereas only a minor impact on *C. elegans* and *C. echinulata* biofilms was observed with the highest concentration used (0.1 ​mg/mL). In contrast, the impact of both molecules on planktonic growth was negligible. 3-Hydroxytyrosol, which regulates biofilm growth in *C. elegans* [17] also inhibits biofilm growth in *C. blakesleeana* when it is present in higher concentrations (0.04 and 0.1 ​mg/mL), but to a lesser degree than *C. elegans*.

**Fig. 5:**
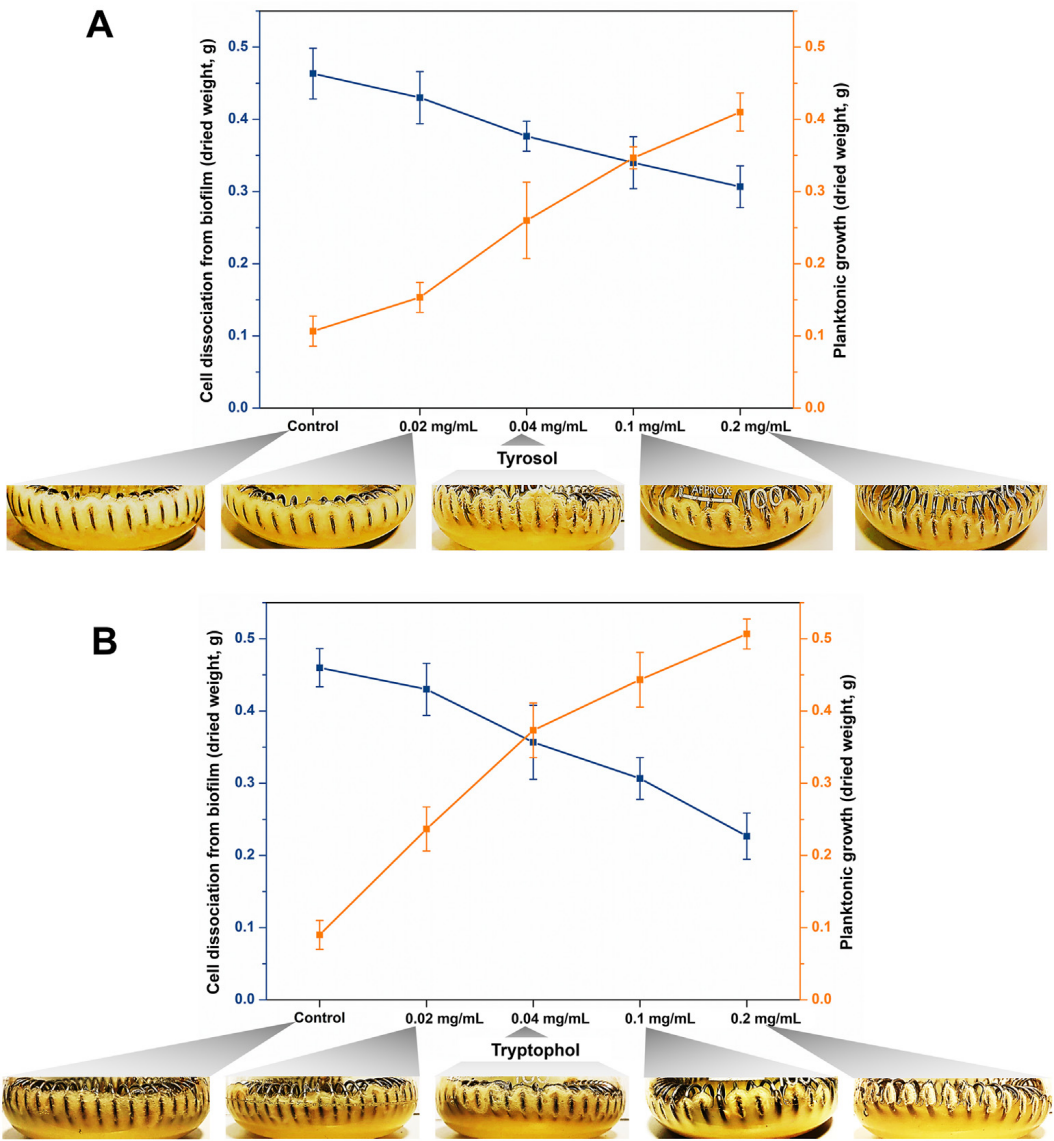
Effect of tyrosol (A) and tryptophol (B) addition to previously grown (144 ​h) ​*C. blakelsleeana* biofilm. The flasks were incubated for a further 96 ​h before the biomass was dried and weighed (all p-values <0.05 for individual concentration of tyrosol and tryptophol when compared with the control cultures to which DMF was added).

Detachment of biofilms can be a result of external factors, such as shearing, or can occur in a co-ordinated manner, in which quorum sensing molecules that regulate gene expression and coordinate cell-cell communication in the biofilm regulate detachment [14,28]. For instance, the exogenous addition of some quorum sensing molecules such as rhamnolipids, lactones or quinolones can cause reduced biofilm growth with induced cell detachment in *Pseudomonas aeruginosa* biofilm [13]. In the present study tyrosol and tryptophol might behave as quorum sensing molecules, thus an experiment was designed to investigate the cell detachment from biofilm of *C. blakesleeana*. To determine if the presence of additional tryptophol or tyrosol had any effect on biofilm that had already grown, *C. blakesleeana* biofilm cultures were grown for 144 ​h and the medium replaced with fresh sabouraud dextrose broth containing different concentrations of either tyrosol or tryptophol and the cultures incubated for a further 96 ​h. As shown in Fig. 5, both molecules caused detachment of biofilm and an increase in planktonic biomass; the degree of change reflected the amount of tyrosol/tryptophol added (R^2^ ​≥ ​0.96). In contrast no detached biomass was visible in flasks of *C. elegans* or *C. echinulata* biofilms (not shown), thus the effect of tyrosol and tryptophol is specific to *C. blaskesleeana*.

## Conclusions

Two molecules, tryptophol and tyrosol, were identified for the first time in culture supernatants of *C. blakesleeana*. Both molecules were present in higher concentrations in planktonic cultures compared with biofilms, and both selectively inhibited biofilm growth and caused detachment of previously grown biofilm. The compounds are known to be produced in yeasts where they have a signalling/quorum sensing role. Tyrosol is produced by the dimorphic yeast *Candida albicans*, where it accelerates hyphal development and counteracts the effects of another QSM, farnesol [26]. [27] demonstrated that treatment of *C. albicans* with tryptophol decreased its virulence and pathogenicity in *Galleria mellonella*. These authors reported an inhibition of yeast-to-hypha transition and increased expression of pro-apoptotic genes in the yeast when tryptophol was present. Tryptophol is produced by *Saccharomyces cerevisiae* under low nitrogen conditions, where it (along with phenylethanol) stimulates morphogenesis in the yeast [11]. Filamentous fungi use quorum sensing molecules for regulating secondary metabolism: for example, the production of the antibiotic sclerotiorin in *Penicillium sclerotiorum* is stimulated by endogenous γ-butyrolactones [25]. Tyrosol is produced by *Penicillium chrysogenum* DXY-1 and was observed to act as an inhibitor of bacterial biofilms [9], but was not examined for its impacts on the producing fungus. Tryptophol has not previously been identified as a signalling molecule in other filamentous fungi.

The production of QSMs by one microorganism can impact the growth of another, for example, exogenously added farnesol and 2-phenylethanol inhibited biofilm growth of the dimorphic yeast *Sporothrix*, whereas biofilm formation was stimulated by the addition of tyrosol [8]. In the present study we investigated the effect of tyrosol and tryptophol on biofilm growth of two other *Cunninghamella* spp., *C. elegans* and *C. echinulata*, which do not produce them. While tyrosol had minimal impact on planktonic and biofilm growth of both fungi, tryptophol selectively inhibited biofilm growth, albeit not to the same degree as *C. blakesleeana*. 3-Hydroxytyrosol, which is produced by *C. elegans* as a biofilm regulatory molecule [17] had a minor inhibitory effect on biofilm growth of *C. blakesleeana*, thus the regulatory molecules identified in *Cunninghamella* spp. thus far are species-specific in relation to their effects on biofilm growth.

In conclusion, we have identified tryptophol and tyrosol as signalling molecules produced by *C. blakesleeana* that regulate biofilm growth. The mechanism of regulation is different from that recently discovered in *C. elegans*, which employs 3-hydroxytyrosol, and *C. echinulata*, for which no signalling molecules have yet been identified. The understanding of biofilm growth control is important since these fungi are important for the production of drug metabolites and degradation of xenobiotics, thus having detailed knowledge of the signalling molecules involved can enable better control of biofilm growth.

## CRediT authorship contribution statement

**Mohd Faheem Khan:** Conceptualization, Methodology, Funding acquisition, Data curation, Formal analysis, Writing – review & editing. **Dua Saleem:** Data curation, Formal analysis. **Cormac D. Murphy:** Conceptualization, Methodology, Funding acquisition, Supervision, Writing – original draft, preparation, Writing – review & editing.

## Declaration of competing interest

The authors declare the following financial interests/personal relationships which may be considered as potential competing interests: Cormac Murphy reports financial support was provided by 10.13039/501100002081Irish Research Council.

## Appendix A Supplementary data

Multimedia componenet 1 Multimedia componenet 1
