# Supplementary material for: Regulation of Cunninghamella spp. biofilm growth by tryptophol and tyrosol
Source: Biofilm. 2021 Mar 26;3:100046. doi: 10.1016/j.bioflm.2021.100046 (PMC8058532; doi:10.1016/j.bioflm.2021.100046)
Supplement: Multimedia componenet 1 [file mmc1.docx]

**Regulation of *Cunninghamella* spp. biofilm growth by tryptophol and tyrosol**

**Supplementary Information**

**
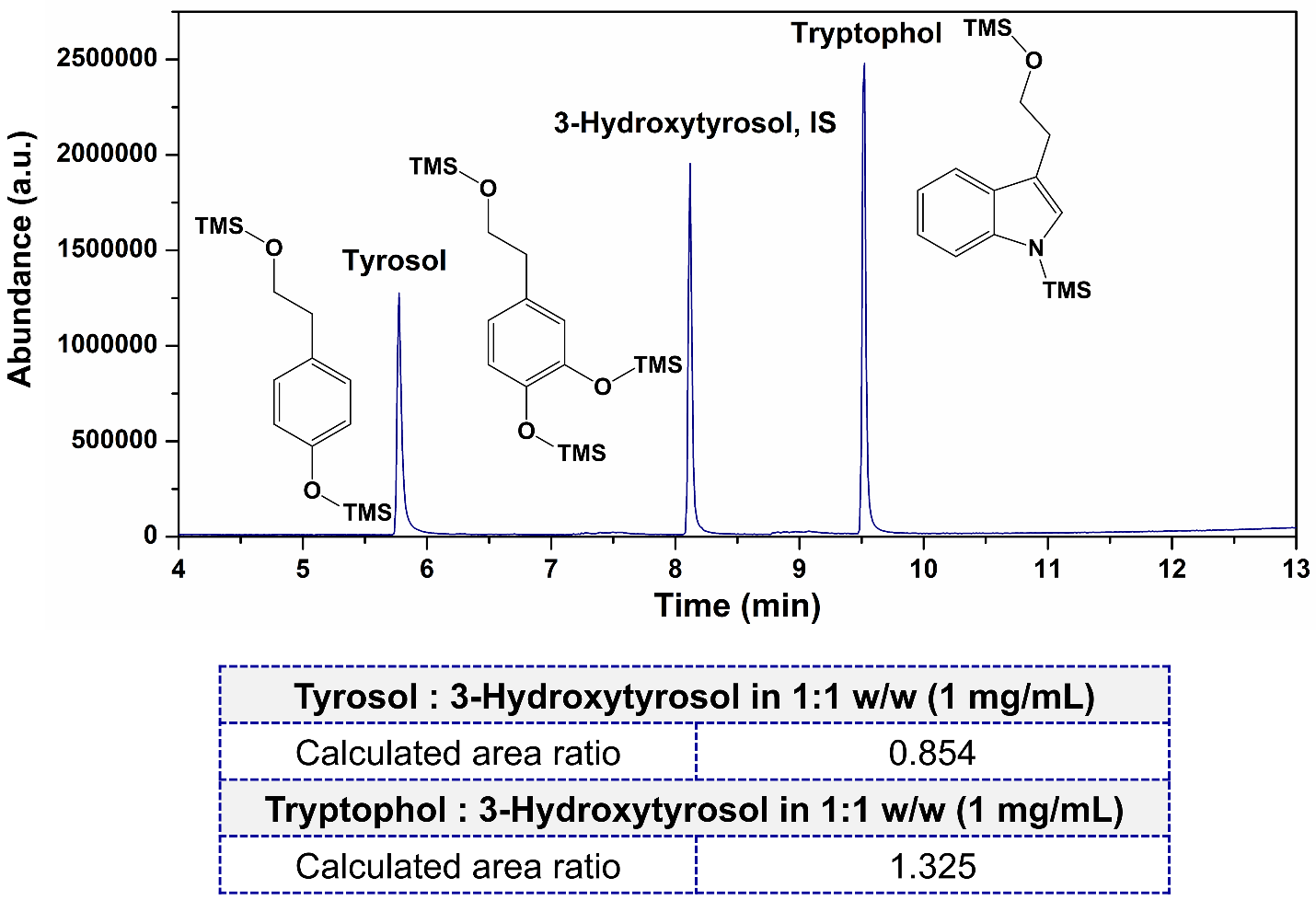
**

**Figure S1:** Total ion chromatograms of standard containing tyrosol, 3-hydroxytyrosol (internal standard, IS) and tryptophol mixed in equal concentration ratio to calculate the factor that used in the quantification of tyrosol and tryptophol production by biofilm and planktonic cultures of *C. elegans.*

**
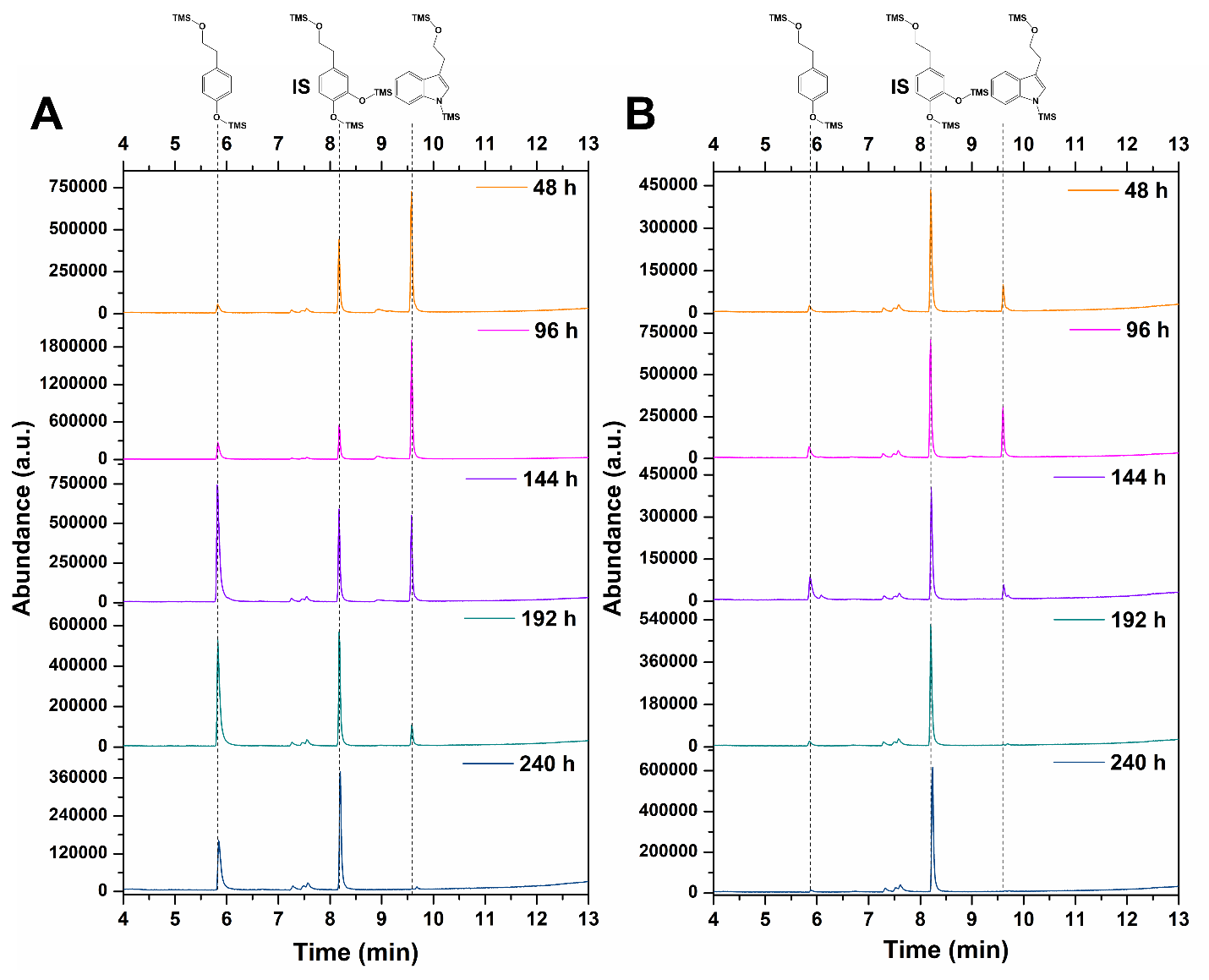
**

**Figure S2:** Total ion chromatograms of culture extracts from *C. blakesleeana* planktonic (A) and biofilm (B) cultures at different times during growth. The internal standard (IS) 3-hydroxytyrosol elutes at 8.20 min and the amount of tyrosol (5.9 min) and tryptophol (9.7 min) were calculated from comparison of the peak areas.

**
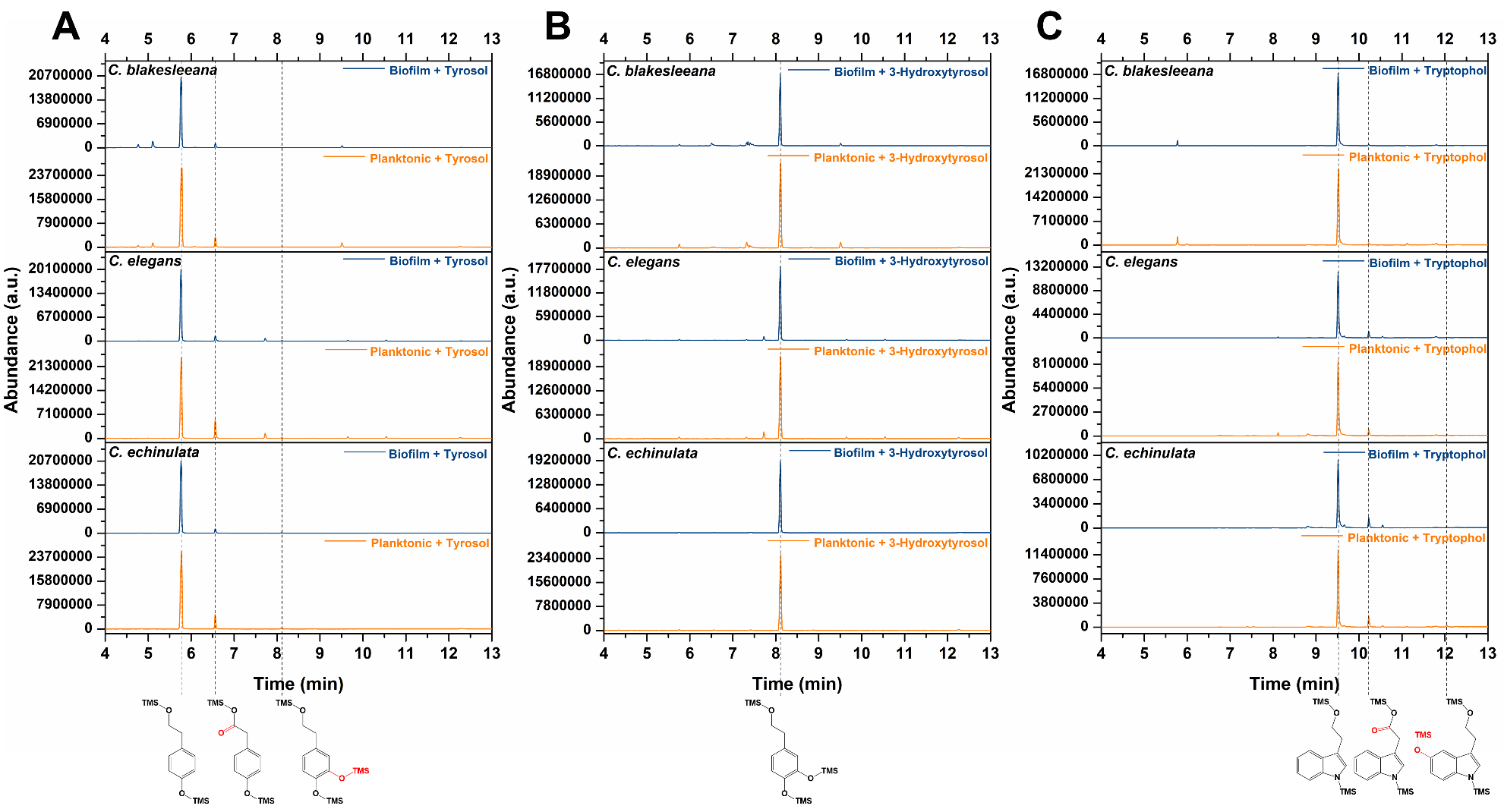
**

**Figure S3:** Total ion chromatograms of culture extracts from biotransformation experiments with tyrosol (A), 3-hydroxytyrosol (B) and tryptophol (C). Minor oxidation products were detected eluting at 6.5 min and 8.1 min in the tyrosol experiment and 10.2 min and 12 min in the tryptophol experiment.

**
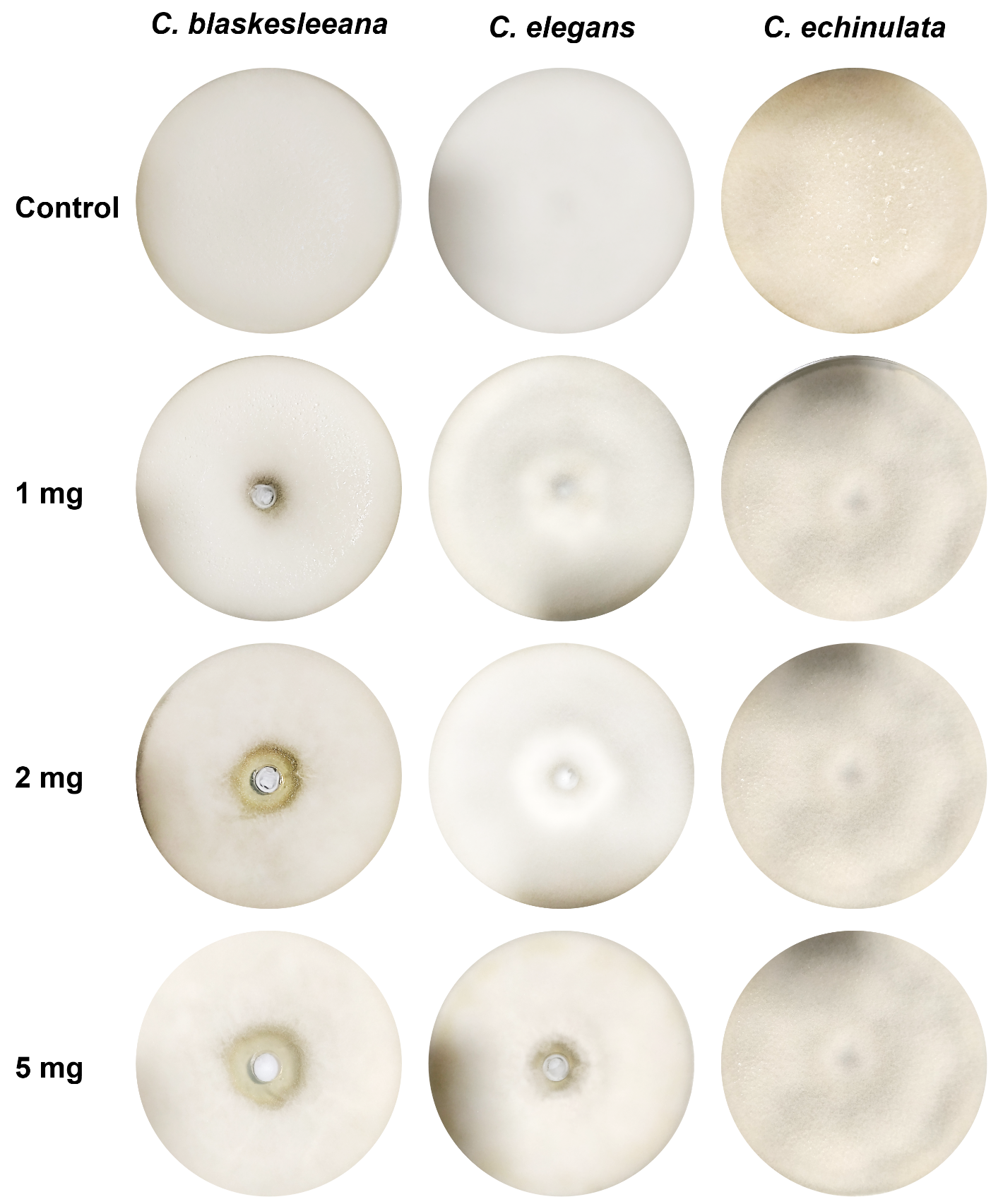
**

**Figure S4:** Agar diffusion bioassay of tyrosol for investigation of its effect on *Cunninghamella* spp. The control plate was treated with DMF only. All plates were incubated for 96 h.

**
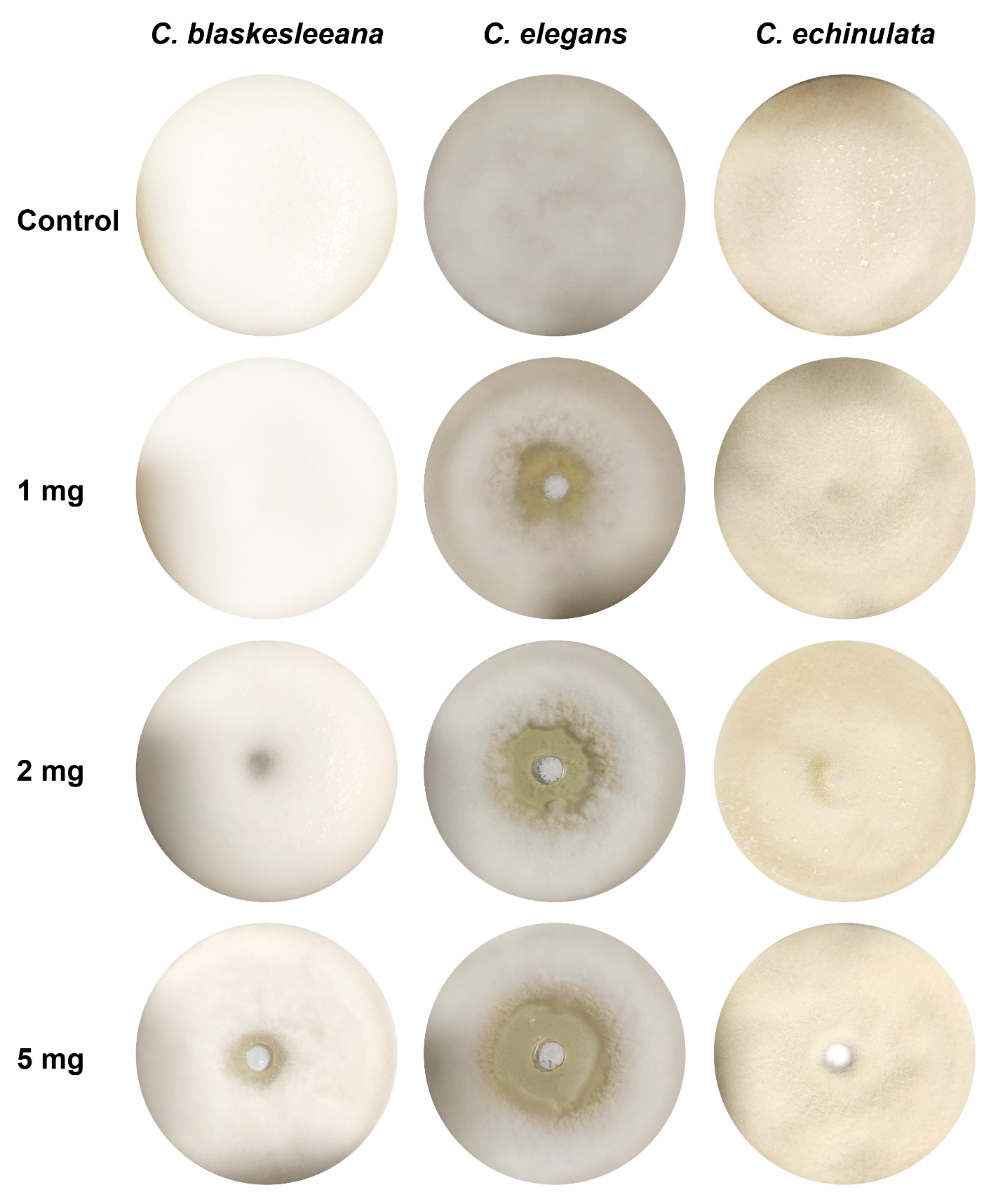
**

**Figure S5:** Agar diffusion bioassay of 3-hydrotyrosol for investigation of its effect on *Cunninghamella* spp. The control plate was treated with DMF only. All plates were incubated for 96 h.

**
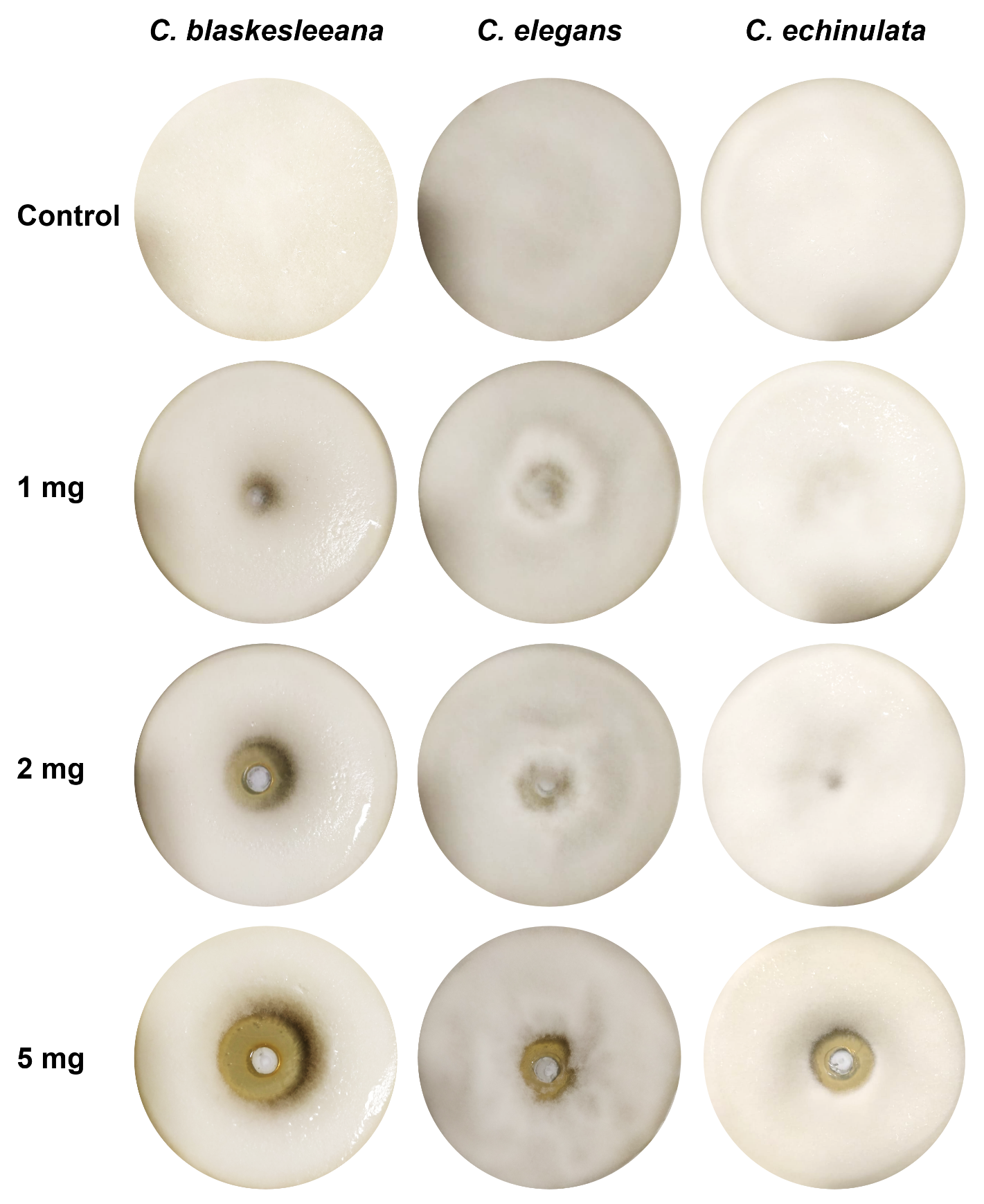
**

**Figure S6:** Agar diffusion bioassay of tryptophol for investigation of its effect on *Cunninghamella* spp. The control plate was treated with DMF only. All plates were incubated for 96 h.
